# Training finger individuation with a mechatronic-virtual reality system leads to improved fine motor control post-stroke

**DOI:** 10.1186/1743-0003-11-171

**Published:** 2014-12-26

**Authors:** Kelly O Thielbar, Thomas J Lord, Heidi C Fischer, Emily C Lazzaro, Kristin C Barth, Mary E Stoykov, Kristen M Triandafilou, Derek G Kamper

**Affiliations:** Sensory Motor Performance Program, Rehabilitation Institute of Chicago, 345 E Superior Street,Suite 1406, Chicago, IL 60611 USA; Department of Biomedical Engineering, Illinois Institute of Technology, Chicago, IL 60616 USA; Department of Occupational Therapy, University of Illinois Chicago, Chicago, IL 60612 USA; Department of Biomedical Engineering, Vanderbilt University, Nashville, TN 37235 USA; Occupational Therapy College of Health Sciences, Rush University, Chicago, IL 60612 USA

**Keywords:** Stroke, Hand, Fingers, Individuation, Occupational therapy, Rehabilitation

## Abstract

**Background:**

Dexterous manipulation of the hand, one of the features of human motor control, is often compromised after stroke, to the detriment of basic functions. Despite the importance of independent movement of the digits to activities of daily living, relatively few studies have assessed the impact of specifically targeting individuated movements of the digits on hand rehabilitation. The purpose of this study was to investigate the impact of such finger individuation training, by means of a novel mechatronic-virtual reality system, on fine motor control after stroke.

**Methods:**

An actuated virtual keypad (AVK) system was developed in which the impaired hand controls a virtual hand playing a set of keys. Creation of individuated digit movements is assisted by a pneumatically actuated glove, the PneuGlove. A study examining efficacy of the AVK system was subsequently performed. Participants had chronic, moderate hand impairment resulting from a single stroke incurred at least 6 months prior. Each subject underwent 18 hour-long sessions of extensive therapy (3x per week for 6 weeks) targeted at finger individuation. Subjects were randomly divided into two groups: the first group (Keypad: N = 7) utilized the AVK system while the other group (OT: N = 7) received a similarly intensive dose of occupational therapy; both groups worked directly with a licensed occupational therapist. Outcome measures such as the Jebsen-Taylor Hand Function Test (JTHFT), Action research Arm Test (ARAT), Fugl-Meyer Upper Extremity Motor Assessment/Hand subcomponent (FMUE/FMH), grip and pinch strengths were collected at baseline, post-treatment and one-month post-treatment.

**Results:**

While both groups exhibited some signs of change after the training sessions, only the Keypad group displayed statistically significant improvement both for measures of impairment (FMH: p = 0.048) and measures of task performance (JTHFT: p = 0.021). Additionally, the finger individuation index – a measure of finger independence – improved only for the Keypad group after training (p = 0.05) in the subset (Keypad: N = 4; OT: N = 5) of these participants for which it was measured.

**Conclusions:**

Actively assisted individuation therapy comprised of non task-specific modalities, such as can be achieved with virtual platforms like the AVK described here, may prove to be valuable clinical tools for increasing the effectiveness and efficiency of therapy following stroke.

**Electronic supplementary material:**

The online version of this article (doi:10.1186/1743-0003-11-171) contains supplementary material, which is available to authorized users.

## Background

Dexterous manipulation of the digits is one of the hallmarks of human motor control. While biomechanical and neurological constraints do limit independent movement of the fingers and thumb to some extent[1, 2], the capabilities of the hand are quite remarkable, far surpassing state-of-the-art robotic hands, for example. Considerable neurological resources are devoted to support hand manipulation, as evidenced by the large representation in both primary motor and sensory cortices[3]. Specialized, phylogenetically recent corticomotoneuronal pathways[4] seem to be necessary for the creation of independent finger movements[5]. These pathways continue to develop postnatally, and do not form synapses with their motoneuronal targets until months after birth[6]. Unfortunately, these pathways may be damaged by neurological incidents, such as stroke. Indeed, individuation can be compromised even when the stroke produces only small[7], or lacunar lesions[8]. The diminished individuation impacts a variety of activities from typing to grasp and transport of objects[9].

Despite the functional importance of hand motor control, relatively few studies have examined the effects of training individuated digit movements post-stroke. Taheri et al., investigated the use of the FINGER robot to train individuation for the middle and ring fingers[10]. Sale et al., examined the feasibility of using a hand-specific robot, the Amadeo® System, in the early phases of stroke rehabilitation[11]. Merians et al., created a set of virtual reality scenarios, including a virtual piano, in order to train the upper extremity after stroke[12]. They observed improvements in a metric they created to assess finger independence. The efficacy of this type of training in comparison to more standard occupational therapy methods, however, is not clear.

We developed an actuated virtual keypad (AVK) system specifically targeting independence of finger movements[13]. Virtual reality was employed as it permits quick, facile alterations in task challenge (e.g., the amount of digit flexion required to play a key) as well as the ability to map different notes to a given key, thereby providing for a limitless set of sounds to be played with a finite set of keys. In this study, we sought to determine whether training with this system would improve independent finger movements and facilitate general task performance in stroke survivors with chronic hemiparesis. We sought to compare the effectiveness of this novel intervention with the outcomes from performing occupational therapy focused on the hand.

While one group trained with a therapist on the AVK, another group received a similar time-dose of intensive occupational therapy focused on fine motor control and finger individuation. We hypothesized that training with the AVK would translate into improved generalized hand motor control to the same extent or beyond that exhibited by the group receiving intensive occupational therapy focusing on the hand. Equivalent success would suggest that the AVK therapy could be employed with multiple users guided by a single therapist or even in remote therapy opportunities to create greater efficiency in the provision of therapy services to this population.

## Methods

### Actuated virtual keypad

**Figure 1:**
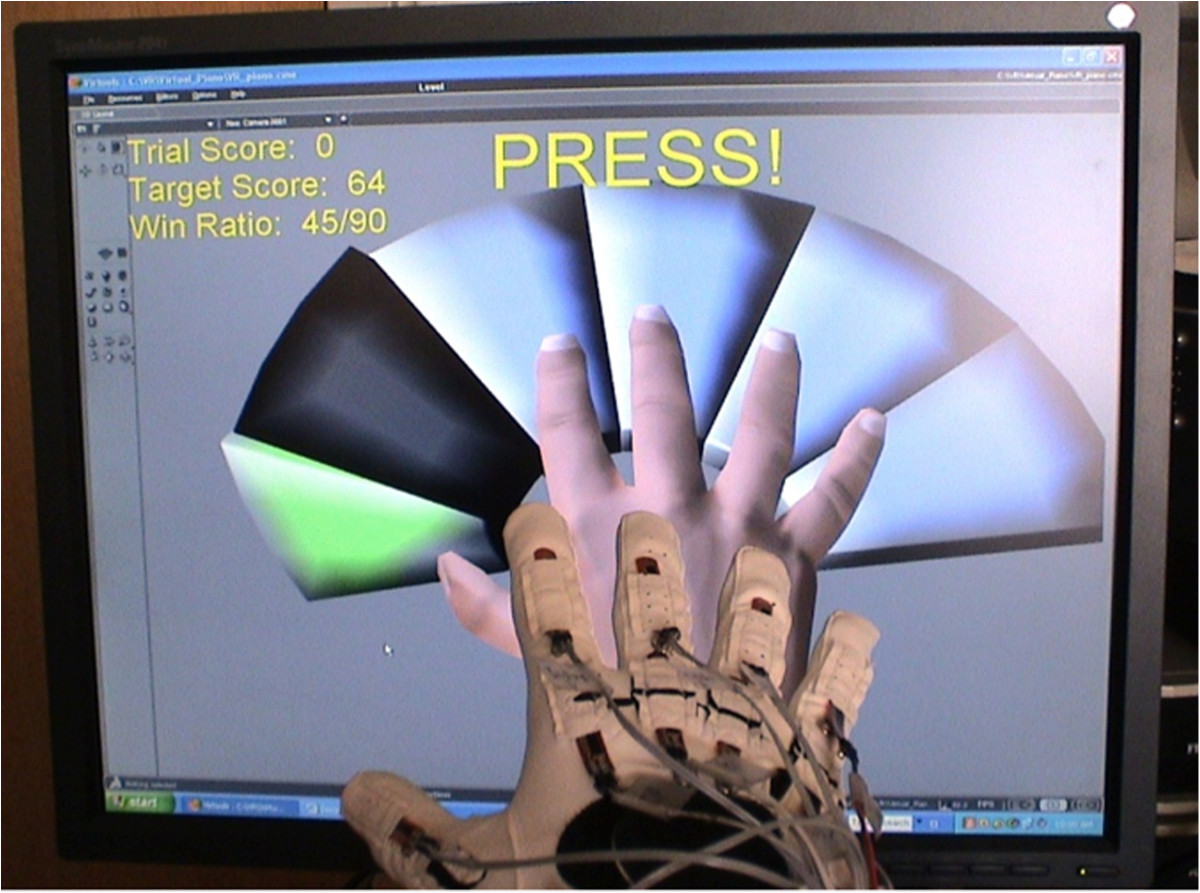
**Actuated virtual keypad (AVK) system.** User wears the PneuGlove which both measures joint angles through bend sensors and provides assistance to finger extension or resistance to finger flexion through pneumatic actuation. The user controls the virtual hand through the PneuGlove, and thus depression of the keys.

**Figure 2:**
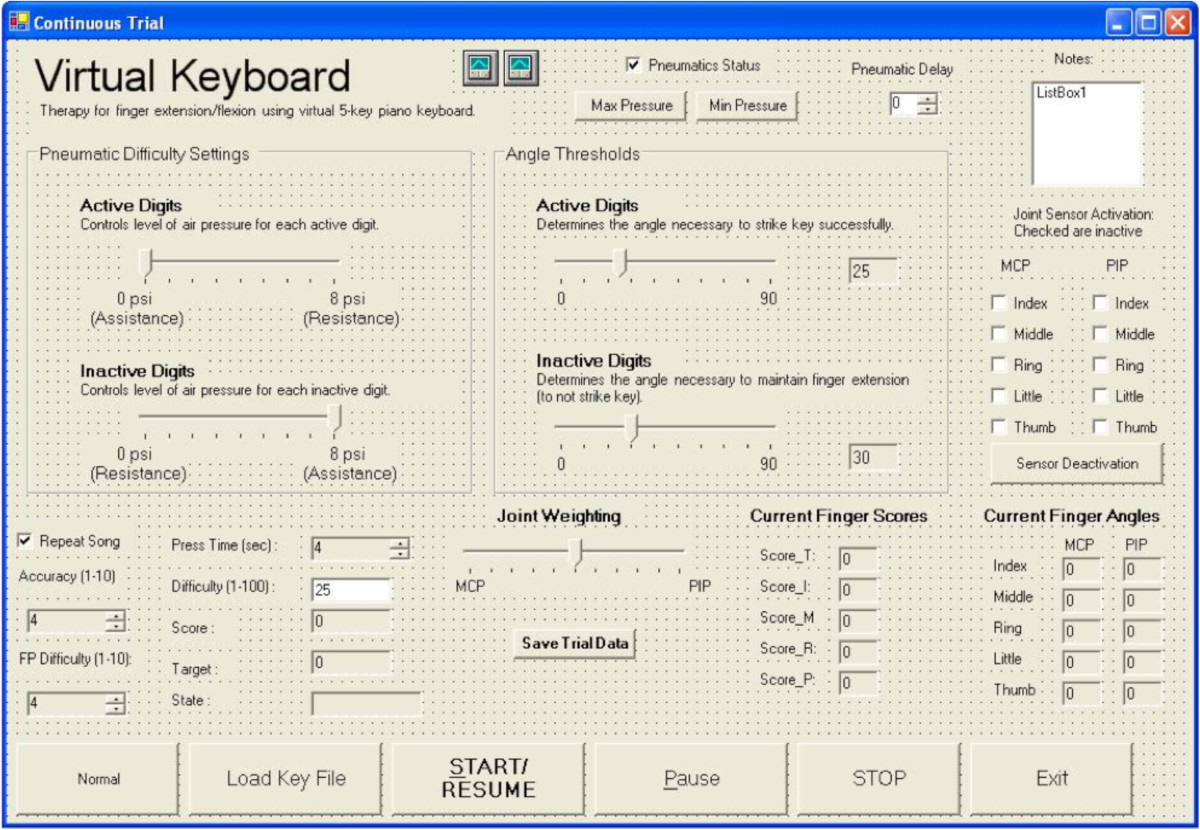
**Graphical User Interface (GUI) for the AVK system.** Therapist adjusts parameters to grade task difficulty according to subjects’ ability level throughout the session, such as the amount of assistance/resistance pressure provided, the angular thresholds for key stroke, and the digits to be monitored.

The AVK system[13] combines a custom actuated glove, the PneuGlove[14], with a virtual scene consisting of a hand and 5 keys. The PneuGlove provides both independent measurement and actuation of each digit. Air pressure, controlled through servovalves (QB1TFEE010, Proportion Air, Inc.) is used to extend (or prevent flexion of) a specified digit by inflating an air chamber located on the palmar side of the digit. Evacuation of the air chamber permits almost unrestricted movement of the digit. Thus, back-drivability is very high. With this manner of actuation, minimal mass is added to the digits; the majority of the 60 g of mass of the glove resides in the connectors located at the wrist. Angles of the metacarpophalageal (MCP) and proximal interphalangeal (PIP) joints of each finger and the MCP and interphalangeal (IP) joint of the thumb are measured with 2-inch bend sensors (2000–0201, Flexpoint Sensor Systems, Inc.) located on the dorsal side of the glove[14].Inputs from the PneuGlove control a virtual scene, created using the software platform Virtools (Dassault Sytemes, France), consisting of a hand and 5 keys (either a left or right hand can be represented, see Figure 1). Posture of the hand is updated in real-time according to the measured joint angles. If the digit is flexed sufficiently, then the corresponding key moves and changes color to indicate that it has been pressed, and a unique tone specific to that key is emitted.Importantly, the system is controlled by a graphical user interface (GUI) which allows the therapist to alter a number of parameters throughout a session in order to guide the treatment (Figure 2). For example, the amount of MCP and PIP/IP flexion needed for a successful key press can be specified, as can the relative weighting on the MCP and PIP/IP angles, varying from all weight being placed on the MCP to a combination of MCP and PIP/IP angles to all weight placed upon the PIP angles. In this manner, the focus of therapy can be adjusted from the MCP to the PIP to a simultaneous flexion of both. The challenge of the task can be adjusted to the capabilities of the user in a number of ways, such as: by adjusting the level of assistance provided by the PneuGlove, by changing the speed at which the keys are to be pressed and released, and by selecting specific key combinations to be practiced.

### Intervention

#### Participants

A total of 16 subjects enrolled in the study. Participants had chronic hemiparesis resulting from a single hospitalized ischemic or hemorrhagic stroke occurring at least 6 months prior to enrolling in the study. Additionally, the participants exhibited mild to moderate hand impairment as evidenced by a score of 5 or 6 on the Stage of Hand subsection of the Chedoke-McMaster Stroke Assessment scale (CMSA-H)[15]. Each participant demonstrated limitations with fine motor control and finger individuation, but was able to perform at least two of the following three movements: 1) abduction of the digits to full range of motion; 2) touching of the tip of the thumb to the tip of the little finger; 3) smooth reversal between full flexion and full extension of the digits. Potential subjects were excluded if they: 1) were receiving outpatient physical or occupational therapy; 2) had biomechanical limitations (e.g., contracture) which limited passive digit extension to 20° of finger flexion; 3) had received a botulinum toxin (e.g., Botox®) injection less than 6 months prior to enrollment, 4) had cognitive deficits limiting simple one-step commands, or 5) had significant upper extremity pain (rated as greater than 6/10). Northwestern University’s Institutional Review Board (Chicago, IL) approved the study design and participants signed written informed consent before enrolling in the study.

#### Protocol

Participants were randomized into one of two treatment groups by drawing lots. Each participant in both groups worked individually with a research occupational therapist for a total of 18 one-hour training sessions 3 times per week for 6 weeks.

**Figure 3:**
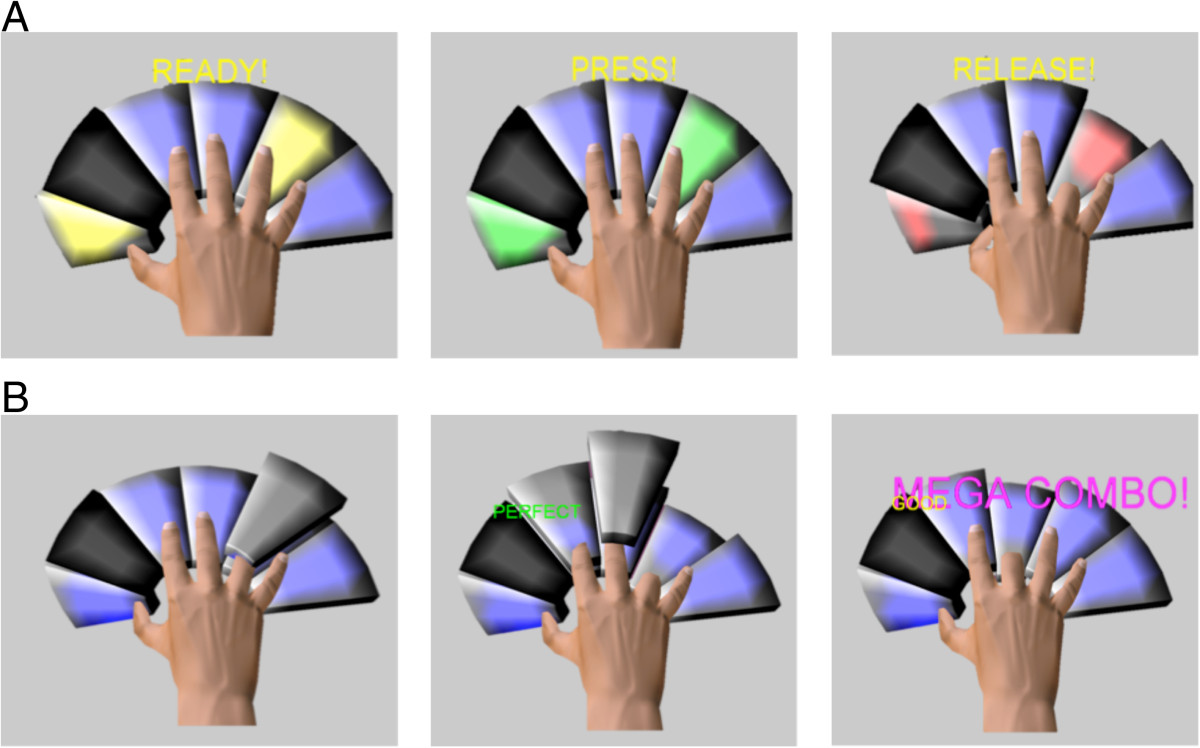
**The AVK system. A)** Key Combination mode—The user must depress the instructed keys within a specified period of time and then release the keys. If unsuccessful, the digits in error are highlighted with red rings at the end of each trial. A score for each trial and a running score are shown on the screen to the user as feedback (not pictured). **B)** Song Mode—The user is given a series of key combinations in order to play the chosen song. The pictured sequence was for the ring finger (first image) followed by the index finger (middle image) and middle finger (last image) independently. A score for each key press was awarded and tallied for an overall song score (not pictured). Bonus points could be earned for perfect sequential key presses (e.g. Mega Combo).

The first group (OT) performed high intensity, task-oriented occupational therapy centered on fine motor control, dexterity, in-hand manipulation, and finger individuation. The task-oriented protocol utilized[16] was developed by one of the authors, Dr. Mary Ellen Stoykov, and she trained the occupational therapists delivering the intervention for this study (see Additional file1). Treatment activities, selected according to the participant’s priorities as determined from the Canadian Occupational Performance Measure (COPM)[17], included practice of buttoning, typing, tying knots, writing, and using tools. In accordance with findings for improving motor learning[18], challenge level of the treatment activity was adjusted to the capabilities of the participant. For example, a therapist could vary the level of external support (e.g., providing or removing tabletop support to grade the activity according to proximal weakness) or the dimensions of the object (e.g., size and mass).The other group (Keypad) trained exclusively with the AVK system to practice movements of different combinations of digits. Two different modes were employed. In one mode, Key Combination, the participant attempted to play the discrete key combinations specified on the computer screen. This involved depressing one or more specified virtual keys while refraining from depressing the others, then holding this key or these keys in the depressed position for a designated amount of time, and then finally releasing them when specified. Visual displays guided the user (e.g., the keys to be played turned green, see Figure 3A), as did the therapist. Visual and audio feedback informed the user of success or failure. For example, the virtual digits creating undesired movements were highlighted with red encirclement. Also, each key was associated with a unique tone, which would play whenever the key was struck. A running score based on performance was displayed to the participant. A computer algorithm kept track of which combinations were most difficult for the participant and adjusted the difficulty of the combinations to the performance of the participant. The PneuGlove assisted in the task, by impeding movement of the unintended digits while permitting free movement of the intended digits during key press and by providing extension assistance during key release.In the Song Mode, participants attempted to play songs, such as "Ode to Joy" as guided on the computer screen in a manner similar to video games, such as Guitar Hero®. Indicators were stacked above the keys to be played at any time point, such that the participant could see the keys to be played a few steps in the future as well as the keys to be played immediately (Figure 3B). The notes first appeared near the top of the screen, above the keypad, and gradually fell down. As the falling note passed the keypad, the participant was expected to strike the corresponding key. Thus the appropriate key (and only that key) needed to be struck and at the appropriate time. Again, the PneuGlove provided assistance. Feedback on timing was provided to the user by displaying "Perfect", "Good", or "Poor" on the screen. Points based on performance, i.e., accuracy and speed, could be accumulated throughout the song and recorded by the therapist. For each note, two points were awarded for "Perfect" timing and one point for "Good" timing, as long as the correct key was played. The acceptable timing threshold range (Good) was set by the therapist through the GUI; a "Perfect" score indicated a timing error occurring within a threshold window that was half as long as the "Good". The score was reset at the end of each song and participants were encouraged to attempt to increase their score each time they played a song. As with the intensive OT treatment, the therapist strove to maintain a proper level of challenge during the Keypad treatment; this was done by manipulating parameters, such as threshold levels of joint flexion for key depression and song speed, on the GUI.

#### Outcome measures

Assessment of the participant’s motor control was performed at three time points during the study: 1) prior to initiation of the training; 2) following the conclusion of the 18 training sessions; 3) one month after completion of all of the training sessions. A research therapist, blinded as to the participant’s group assignment, administered a battery of evaluations. The clinical outcome measures consisted of the Action Research Arm Test (ARAT)[19], the Jebsen-Taylor Hand Function Test (JTHFT)[20, 21], the Upper Extremity Portion of the Fugl-Meyer Motor Assessment (FMUE) and the Hand subcomponent (FMH)[22]. Additionally, the following measurements of strength were also performed: grip strength (GS) (JAMAR 5030 J1 Hand Dynamometer), lateral pinch strength (LPS) (PG-60, B&L Engineering), and 3-point pinch strength (PPS) (PG-60, B&L Engineering).

Finally, we were able to obtain measurement of kinematic individuation for a subset of the participants in each group. The CyberGlove (CyberGlove Systems, LLC, San Jose, CA) was used to measure flexion/extension angles at each digit joint simultaneously. Initial position was a neutral posture for the forearm (0 degrees supination or pronation) and the digits. Participants were asked to move each digit independently throughout their range of motion, beginning from a neutral posture and proceeding through a fully extended posture to a comfortably flexed posture and back to neutral. Subjects were instructed to move at a comfortable speed (up to 10 sec per finger per trial) to minimize movement in the alternate digits. Wrist movement was prevented through splinting (FUTURO™, 3M) which was worn under the CyberGlove. Five successful trials were recorded per digit (25 total).

#### Data analysis

Total scores for each assessment period for each participant were used for the ARAT, FMUE, and CH. The mean score for the three trials taken during each assessment session were averaged to obtain the measures for maximum GS, LPS, and PPS. In accordance with other studies[12], we did not include the handwriting portion of the JTHFT in our outcome measure. Scoring for this item is dependent upon which hand is dominant and which hand is impaired. In this study we do not differentiate hand dominance and side of impairment in analysis; therefore it was not appropriate to include this item in analysis. The total completion time across the other 6 tasks was used.

A variety of kinematic measures have been used to compute individuation indices for the digits[1], including slopes of the displacements[23], fingertip displacement[24], and MCP rotation[8]. We observed that the amount of movement in the intended digit could substantially impact the amount of movement observed in the unintended digits. We also observed that different subjects used different movement strategies, with some favoring greater PIP flexion and others favoring more MCP flexion. Thus, we chose to employ a metric in which we examined the combined MCP + PIP (MCP + IP for thumb) angular displacement of the two joints for each digit up to the point at which a total of 90° of flexion was achieved in each digit. Thus, for the computation of the individuation index[1] shown in Eq. 1, the parameter (S_ij_) term consists of the combined joint angles for each digit.

1 $$II_{j}=1-\left[\left(\sum_{i=1}^{n},∥S_{ij}∥\right)-1\right]/\left(n-1\right)$$

Statistical analyses were performed separately for each group to determine whether the training impacted the outcome measures. Non-parametric Friedman Test for repeated measures was employed due to the ordinal nature of much of the data and the relatively small sample sizes. If the main effect of evaluation session were found to be significant, post-hoc Wilcoxon signed-ranks tests were subsequently performed to determine significant differences between the three different evaluations (pre-treatment, post-treatment, and one-month follow-up). For the individuation indices, paired t-tests were utilized to compare the metrics across all digits from pre- to post-treatment.

To ascertain the effectiveness of the AVK treatment compared to the intensive hand therapy, noninferiority testing was performed as our between-group analysis[25]. The inferiority margin, δ, was set according to the published minimally important clinical difference (MICD) in current literature when available (ARAT[26], FMUE[27], GS[28]) and conservatively estimated for LPS and PPS based on reported minimal detectible changes (MDC)[29]. As the JTHFT has neither the MICD nor the MDC established, δ was estimated according to a 20% improvement from mean baseline scores[12]. In cases for which the AVK treatment showed superiority to the OT treatment, *post hoc* t-tests were performed. To compare the baseline values for the outcomes between the groups, the Kruskal Wallis Test (KW) was used.

## Results

A total of 14 subjects (7 in each group) completed the training and all three evaluation sessions; one participant (included in the analyses) completed 17 of 18 treatment sessions due to scheduling conflicts. Two subjects were forced to drop out before completing the study, one because of unrelated medical issues and one because of personal scheduling conflicts. Data from these subjects were not included in the analyses. Of the subjects completing the study (9 male/ 5 female), the mean age was 57 (range: 46–70) years and the mean time post-stroke was almost 4 years (range: 6–136 months, see Table 1). The two treatment groups were well matched in terms of age, hand impairment, and time post-stroke (Table 1). Age (KW: p = 0.20) and months post-stroke (KW: p = 0.95) were not significantly different. Additionally the initial values describing motor control, FMUE (KW: p = 0.09), FMH (KW: p = 0.27), JTHFT (KW: p = 0.61), and ARAT (KW: p = 0.73), were not significantly different between the groups.

**Table 1:** **Subject characteristics**

Subject	Chedoke	Sex	Age	Months Post Stroke	Affected Arm
**Keypad**					
**E1**	6	F	53	36.2	R
**E2**	5	M	57	96.3	R
**E3**	5	F	51	6.6	L
**E4**	5	M	46	61.2	L
**E5**	5	M	56	12.6	R
**E6**	5	M	49	39.4	L
**E7**	5	F	69	74	R
**Mean (SD)**	5.1 (0.4)	57.1% Male	54 (7)	46.6 (32.5)	42.9% left affected
**OT**					
**C1**	5	F	58	89	R
**C2**	5	M	58	10.2	L
**C3**	5	M	70	14.2	L
**C4**	5	F	53	136.4	L
**C5**	5	M	56	15.4	R
**C6**	5	M	66	35.3	L
**C7**	5	M	55	35	L
**Mean (SD)**	5.0 (0)	71.4% Male	59 (6)	47.9 (47.4)	71.4% left affected

**Figure 4:**
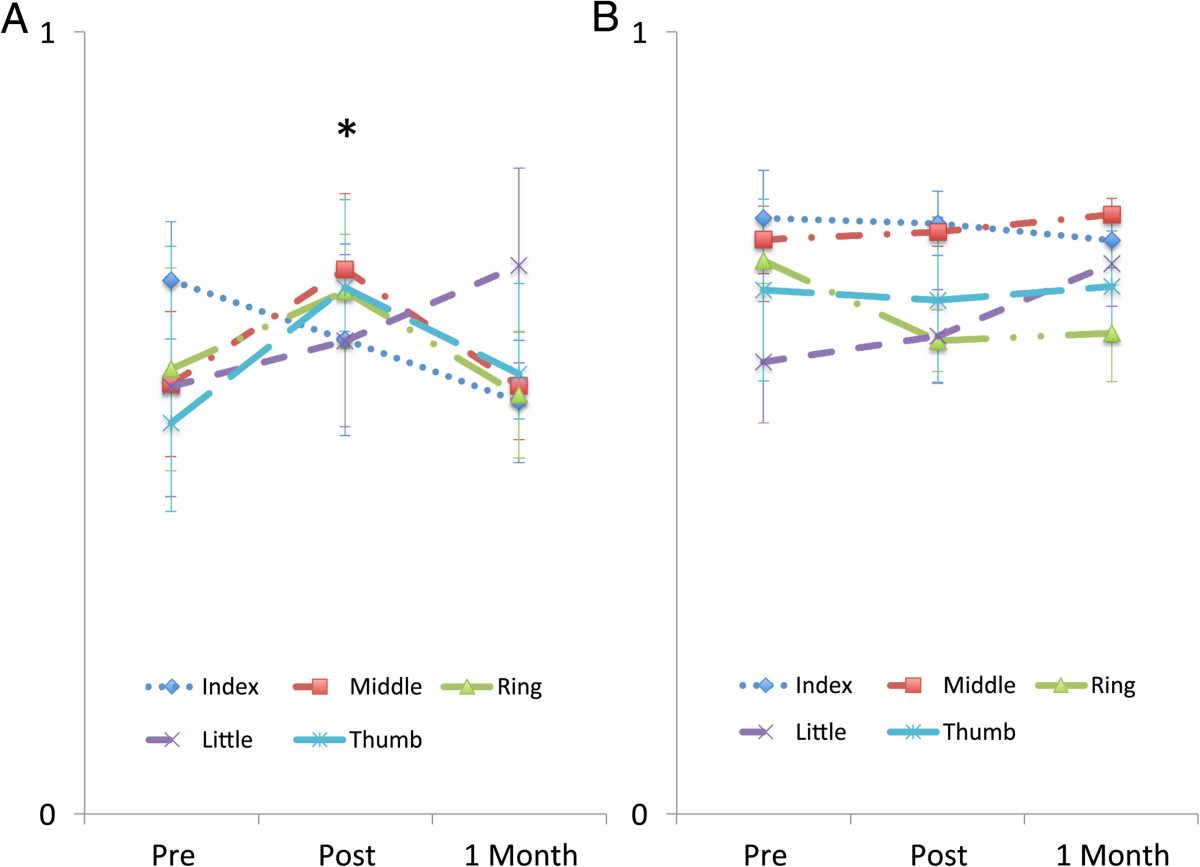
**Change in finger individuation.** Values averaged across subjects for each digit for **A)** AVK **B)** OT. Error bars represent SE. *Paired t-test: p-value = 0.050 across all digits.

On average the OT group completed 267 (14) task movements with a range of 230–324 for each training session. There were some trends for improvement in the OT; notably mean FMUE score increased by 3.1 (5.9) points from baseline to follow-up as did the FMH subtest which increased by 1.7 (2.6) points over the same period (Table 2). Although mean grip strength did improve by 25 N, or 12% from baseline to follow-up, no statistically significant changes were exhibited after training for any of the outcome measures for this group (Friedman Test: p > 0.05). The individuation index remained quite constant for the OT group subset, with average values of 0.69 (0.16), 0.67 (0.14), and 0.70 (0.11) (paired t-test: p = 0.51) across the three evaluations (Figure 4B). Similarly the ratio of MCP to PIP flexion did not change greatly for this group; MCP flexion changed from 42% to 45%, pre-evaluation to the one-month follow-up.

**Table 2:** **Values of outcome measures and within-group analyses**

Outcome measure	OT				AVK			
	Pre	Post	Follow-up	p-value	Pre	Post	Follow-up	p-value
FMUE	41.9 (1.9)	43.6 (8.1)	44.9 (7.2)	0.717	48.7 (9.6)	50.4 (10.4)	50.0 (8.7)	0.048*
FMH	12.9 (3.0)	13.4 (5.7)	14.6 (3.1)	0.580	15.4 (5.0)	16.9 (5.5)	17.3 (4.9)	0.340
ARAT	48.1 (7.7)	44.6 (12.7)	45.3 (11.2)	0.895	46.6 (8.9)	49.7 (8.8)	51.4 (7.0)	0.142
JTHFT	128.3 (67.4)	127.7 (67.8)	145.9 (92.0)	0.651	108.3 (74.5)	103.7 (88.8)	75.3 (50.1)	0.021*
GS (N)	191 (72)	200 (59)	214 (67)	0.051	250 (84)	275 (100)	255 (93)	0.368
LPS (N)	75.6 (33.4)	66.7 (9.5)	69.3 (16.0)	0.607	83.2 (34.9)	81.0 (27.1)	83.5 (32.9)	0.867
PPS (N)	53.6 (26.4)	51.5 (9.1)	53.2 (8.5)	0.513	61.4 (28.4)	58.9 (28.0)	58.6 (29.1)	0.772

Values are Mean (SD) and *indicates significance for an α-level of 0.05. P-values are results from the Friedman Tests. No significant difference was observed between the baseline measures for any of the outcome measures (Kruskal Wallis Test p > 0.142).

In contrast, the Keypad group completed almost 1000 key presses each session (range 750–1200), with the majority of key presses occurring during the Song mode. Subjects in this group showed significant improvement on multiple measures between baseline and the one-month follow-up (Table 2). Improvements were observed both on measures of impairment and measures of performance. The FMH score improved by 1.9 (1.5) or 12% (p = 0.026). The JTHFT showed an average decrease of 33 seconds for the time to complete the 6 tasks (p = 0.028). The ARAT also showed improvement, with a score increase of 4.8 (7.2). Improvement in digit individuation was also apparent in the subset of Keypad group members who performed these tests (Figure 4A). Despite the downward trend observed for the index finger, the average individuation index across all digits increased from 0.57 (0.23) to 0.65 (0.20) (paired t-test: p = 0.05) for these participants, although it decreased back to 0.57 (0.18) at the one-month follow-up. Interestingly, there was a shift toward greater MCP flexion to perform the movement, from 39% at the pre-evaluation to 49% at the one-month follow-up.

**Figure 5:**
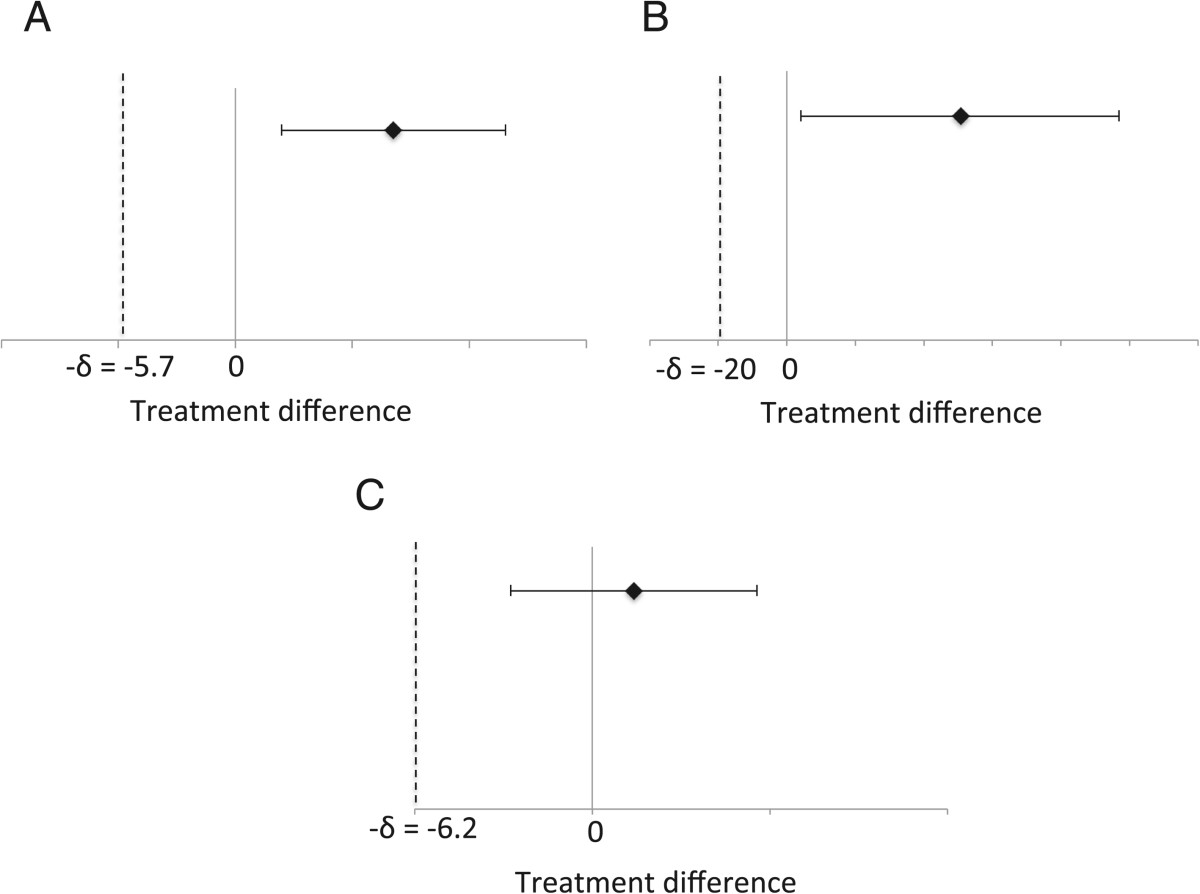
**Noninferiority testing of treatment difference (AVK-OT) at one-month follow-up relative to baseline. A)** ARAT and **B)** JTHFT both showing AVK superiority and **C)** LPS showing noninferiority.

**Figure 6:**
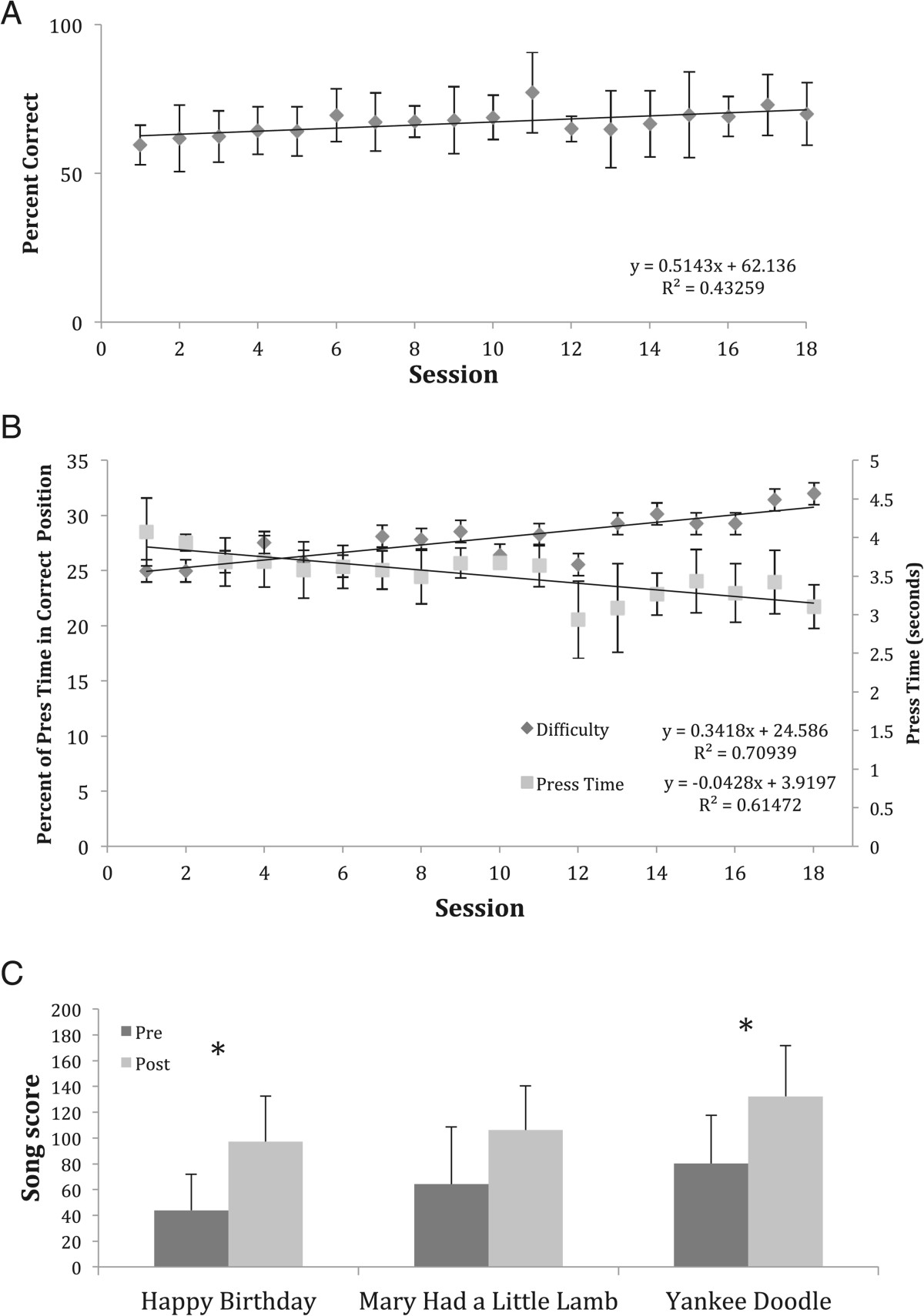
**Performance across training sessions on exercises with the AVK system.** Success in Key Combination Mode as quantified by the **A)** Win Ratio and **B)** Difficulty. Markers indicate mean across subjects for each session. **C)** Change in performance of Song Mode for a single subject. Comparison of mean song score during the first (dark gray bar) and last session (light gray bar) for a single subject using the same difficulty settings. Error bars indicate SD. *p-value < 0.005.

Noninferiority testing confirmed AVK treatment was not inferior to the intensive OT treatment in promoting changes for any of the outcome measures of this study. In fact, AVK treatment was superior to the intensive OT treatment for two measures, ARAT and JTHFT (Figure 5). Subsequent *post hoc* t-tests revealed a significant difference between groups on the ARAT (p = 0.022) and a trend approaching significance for the JTHFT (p = 0.068). Subjects in the AVK group were able to improve their ARAT scores by 4.9 (7.2) from pre-treatment to follow-up while the subjects in the OT group actually showed a mean decrease of -2.9 (5.5) over the same time period. Similarly, the AVK subjects were able decrease the time needed to complete the JTHFT by 33.0 (50.9) s while the OT subjects showed a mean increase in time of -17.0 (66.4) s.Participants in the Keypad group were generally able to improve their performance across training sessions on exercises with the AVK system. Despite the fact that the task difficulty level, quantified by the percent of the available time (Press Time) during which the subject was required to maintain the correct finger posture, increased while Press Time decreased (requiring faster response time), the success (as measured by the Win Ratio) remained constant, or even slightly improved (Figure 6A and B) over the training sessions. Individual subjects also displayed improvement on performance of a particular song (Figure 6C).

## Discussion

This preliminary study demonstrated the feasibility of using the AVK system to train fine finger movements. Participants tolerated the system sufficiently that they were able to complete the training sessions without premature withdraw or non-compliance. More importantly, statistically significant changes in multiple outcome measures were observed in this study group, both for measures of impairment (FMH) and measures of task performance (JTHFT). Additionally, change in scores on the ARAT approached the Minimal Clinically Important Difference (MCID) of 5.7[26] even though our population had a high baseline score which sometimes resulted in a ceiling effect. MCID values have not been established for the other outcome measures employed, but the decrease in time to complete the JTHFT was substantial (30% of the pre-training time). Intriguingly, members of this group also exhibited improved digit individuation, aside from the index finger, following training. The increase in the individuation index was not maintained at follow-up, but the change in strategy favoring greater MCP rotation relative to PIP rotation during individuated finger flexion was maintained. The new ratio could place the digit in a more functional posture during closing[30].

Improvement in all outcomes was at least as great for the group using the AVK system as for the group receiving intensive, targeted occupational therapy. In fact, for the two measures most closely assessing hand motor control – ARAT and JTHFT – improvements were significantly greater or approaching significance for the group using the AVK system. This was apparent despite the relatively small subject numbers. Outcomes may have been generally better for the Keypad group using the AVK due to the greater number of movements performed. While the OT group practiced a wider variety of motor skills including reaching, grasp-and-release, finger and wrist activities, and fine motor tasks, the Keypad protocol encouraged more repetitions of the same movement task which required constant finger individuation. Indeed, many more key presses (on average 270% more or 700 presses) could be performed, especially in the Song mode, than task-oriented movements completed by the OT group. Merians et. al. and Hesse et. al. have surmised that movement number was a contributor to improvements they observed[12, 31]. Additionally, it is possible that subjects with more mild hand impairment derive greater benefit from intensive practice of distinctive tasks that require continuous refinement of a specific motor skill. Although individuation exercises and tasks were included in the OT protocol, they were under a larger umbrella of tasks addressing the arm, wrist, and fingers both separately and together. Finally, the assistance provided by the PneuGlove may also have contributed to the improvement. By impeding undesired movement of the uninstructed digits, the device allowed users to focus more attention on proper movement of the instructed digits while still providing appropriate proprioceptive feedback.

Interestingly, the Keypad group exhibited a significant decrease in the time needed to complete the manipulation tasks that comprise the JTHFT. Thus, even though none of the tasks practiced during the Keypad treatment (which included no manipulation of real objects) resembled the tasks required in the JTHFT, performance improved considerably after the treatment. This is in agreement with the study of Merians, et al., who observed decreased times for the JTHFT after performing a variety of virtual exercises unrelated to the JTHFT[12]. Together, these results further support the observations of Schaefer, et al., who found generalization in improvement in unpracticed motor tasks after training in other tasks[32]. This generalizability seemingly results from improved motor control rather than learning of a specific task.

Certainly, there were limitations to the generalizability of the results of this study. Overall this preliminary study had relatively low subject numbers that prevented more robust analyses such as repeated measures ANOVA. The initial FMUE scores were slightly higher on average (although not significantly different) for the Keypad group (48.7 ± 9.6) than for the OT group (41.9 ± 1.9). The difference in outcomes between the groups, however, did not appear to arise from differences in initial impairment levels. Little correlation was seen between initial FMUE and change in JTHFT, for example, for either group (Keypad: r = -0.08; OT: r = -0.19). Additionally, initial hand impairment, as measured by the CMSA-H, was closely matched for both groups. All participants were at CH 5 except for one CH 6 in the Keypad group; this subject at CH 6 showed little change with evaluation session across outcome measures. The individuation analyses were performed only on a subset of participants due to technical difficulties. Future studies examining the generalizability of the findings here, such as a randomized controlled trial, are warranted.

The AVK system promotes usage of a variety of digit movements. Certainly some of these movements (e.g., isolated movement of the ring finger) are used much less often than other movements (e.g., movement of the index finger and thumb together) in activities of daily living. Yet, we feel it is important to attempt a variety of tasks in order to explore the movement workspace. For example, a small study was conducted in our laboratory to examine the impact of training pinching forces in the index finger and thumb[33]. Stroke survivors often have difficulty properly directing these forces, such that excessive shear force is created and the object slips from their grasp. Participants were asked to create forces in a variety of directions, not only in the functionally important normal direction. After only a couple of training sessions, pinching force direction improved significantly. These results, together with the results of the current study, suggest that interventions which encourage exploration of all regions of the workspace may be beneficial for rehabilitation.

## Conclusions

In summary, training of individuated digit movements was performed with a novel system using a multisensory virtual keypad working in conjunction with an actuated pneumatic glove. Stroke survivors with chronic impairment were able to successfully use the system to improve hand motor control. Thus, repetitive movement therapy for independent finger movements, such as with AVK system, may be beneficial and warrants further exploration.

## Electronic supplementary material

Additional file 1: Modified Occupational Therapy Task-Oriented Training Protocol for the Upper Extremity. (PDF 115 KB)
