# Supplementary material for: Training finger individuation with a mechatronic-virtual reality system leads to improved fine motor control post-stroke
Source: J Neuroeng Rehabil. 2014 Dec 26;11:171. doi: 10.1186/1743-0003-11-171 (PMC4292811; doi:10.1186/1743-0003-11-171)
Supplement: Supplementary file 1 — Additional file 1: Modified Occupational Therapy Task-Oriented Training Protocol for the Upper Extremity. (PDF 115 KB) [file 12984_2014_690_MOESM1_ESM.pdf]

APPENDIX 1 – Modified Occupational Therapy Task-Oriented Training Protocol for the Upper Extremity

<table>
<tr><th colspan="2">Prefunctional Activities</th></tr>
<tr><td>Distal Exercises

1. Individual finger flexion and extension exercises (may include resistance or blocking)
2. Finger abduction/adduction (may include resistance)
3. Opposition of thumb to all digits, with isotonic holds using objects between thumb and digit
4. Lift fingers off table individually
5. Practice finger tapping - use all four fingers and then one at a time
6. Increase strength of intrinsics (lumbricals, abductors, and adductors) as needed. Start with placing hand in position and asking subject to hold.
7. Make a fist and then straighten one finger at a time as if you were counting</td><td>Wrist/Forearm Exercises

1. Place and hold from forearm neutral to various degrees of supination/pronation
2. Isotonic supination and pronation with isometric holds at end ranges
3. Isotonic supination/pronation with graded resistance
4. Practice performing figure eights on a baton or dowel rod</td></tr>
<tr><th colspan="2">Graded Activities to Promote Grasp, Pinch and Release</th></tr>
<tr><td>Grasp and Pinch

1. Strengthen thumb in radial abduction to increase stability
2. Subject opens hand to grasp and hold object, moves thumb up and down object surface while maintaining grasp, then releases object on table top
3. Subject opens hand to grasp small pill bottle in hand and uses thumb to lift lid while maintaining grasp, then release bottle onto table top
4. Grasp object on table that is supported by therapist (therapist is holding/stabilizing the object)
5. Grasp bean bag on table
6. Crumple up a sheet of newspaper into a small ball with one hand
7. Grasp object without support by therapist
8. Grasp object with eyes closed (may help increase hand opening)
9. Train grasp/pinch using a variety of objects
10. Grasp an object and then change the weight of it (e.g. grasp a cup and therapist can pour liquid into it or subject can pour with unaffected hand)
11. Train to grasp an object and then rotate it (e.g. pick up box of cereal and then rotate box to pour cereal in bowl)
12. Work on repetitive grasp and release of one object increasing speed
13. Grasp object without support by therapist
14. Grasp object with eyes closed (may help increase hand opening)
15. Train grasp/pinch using a variety of objects
16. Hold onto jar with unaffected hand and unscrew lid with affected. Vary diameter of jars and work up to peanut butter jar.
17. Grasp an object and then change the weight of it (e.g. grasp a cup and therapist can pour liquid into it or subject can pour with unaffected hand)
18. Work on reaching maximum grip or pinch as fast as possible using dynamometer or pinch gauge
19. Apply resistance to wrist extensors while subject is holding/grasping object. Ask subject to hold
20. Place palm flat on table. Raise and lower each finger at one time.
21. Train to pour water from one container to another. Starting at tabletop and then progress to free space. Also, vary size of container. Start with a large glass and progress to pouring with small glasses. Vary the quantity of liquid.
22. Work on repetitive grasp and release of one object increasing speed
23. Strengthen grip and pinch with Theraputty or other resistive equipment</td><td>Release

1. Release of a medium width cylindrical object (can or glass) that is externally supported (on table or countertop) and with the therapist's hand stabilizing the object. Therapist's hand should be on top of the can while the subject attempts to release his/her hand, which is gripping sides of can. Therapist maintains hold on can while subject releases. Task will be easiest at this level since less force modulation is required for this task.
2. The same as above with different object widths
3. Same as above using grasp with forearm in neutral
4. Release object on to a moving target (rolling cart). Subject is standing. Therapist is walking with cart
5. Release unsupported object free in space
6. Release of small ball onto floor using increased velocity
7. Release of objects into progressively smaller spaces
8. Using both hands, grasp/release a dowel using hand over hand (up/down & crossovers)
9. Release of objects using two targets of different height (e.g. shelves)
10. Transport an object and then release
11. Release object into crowded environment without changing position of other object
12. Place a stack of plastic cups upside down on the table. Grasp top cup and turn it over
13. Train release with increasing speed requirements
14. Train release of objects after orientation of hand has to change (thumb facing ceiling while putting mail in mail lot).</td></tr>
</table>

Activities to Promote Reach to Grasp Sequence

1. Work on reaching to target which is visible but with no vision of the arm
2. Work on reaching to various targets using slow movements interspersed with fast reaching movements
3. Work on reaching across midline (using shoulder adduction) and grasping an object with arm free in space and release
4. Work on reaching in sagittal plane (arm in line with shoulder) and grasping an object with arm free in space and release
5. Work on reaching away from midline and grasping an object with arm free in space and release
6. Reach/grasp a moving object from a rolling cart (Subject is standing or walking. Therapist rolls cart with objects and subject grabs object from cart)
7. Participant is in sidelying or standing and holds a paint roller and then moves it up the wall while depressing the scapula. Can begin with active assist.
8. Practice reaching and turning on a light switch
9. Work on reaching slowly and having subject attend to thumb position after receiving information on target (vision of target occluded)
10. Work on moving the arm through a maze of objects while holding an object
11. Train to reach and grasp an object from areas with many objects without knocking over these other objects (e.g. kitchen cabinet, refrigerator or table with many objects placed near the one which is the target object)
12. Reach/grasp with increasing speed
13. Place a stack of plastic cups upside down on the table. Grasp top cup and turn it over.

Fine Motor Activities

Three Jaw Chuck	Lateral Pinch	Pincer Grasp	Tripod
1. Hold sugar packet with affected hand; practice opening packet<br>2. Practice picking up a small salt shaker or a salt packet with three jaw chuck prehension pattern, then turn over and gently shake<br>3. Pick up blocks/checkers/dice and release.<br>4. Grasp, pinch and release large clips on the edge of a box.	1. Practice pouring from a container with a handle (tea kettle or pitcher)<br>2. Practice placing envelopes in simulated mail slot on box<br>3. Set a table using affected hand to place flatware<br>4. Practice counting bills while holding bills with unaffected hand<br>5. Practice putting key into lock<br>6. Practice opening door with key<br>7. Practice zipping and unzipping on dressing board, clothing, pocket book, etc.<br>8. Practice unwrapping thread around spool using affected hand to hold thread (can downgrade by using yarn)<br>9. Play cards – shuffling and dealing<br>10. Pick up a card from edge of a raised surface/box and release<br>11. Practice opening up containers with small lids (soda bottles)<br>12. Hold and drink with cup with handle<br>13. File nails with emery board<br>14. Cut with large scissors<br>15. Cut with small scissors<br>16. Pinch, place and release close pins<br>17. Practice pulling cap off pen<br>18. Use butter knife to cut bread (can substitute Theraputty) Can also spread butter on bread	1. Turn pages of photo album, progress to newspaper or magazine<br>2. Place/Remove pegs to/from pegboard (small ones)<br>3. Pick up pennies from table<br>4. Put coins in slot on simulator box<br>5. Stack coins on table<br>6. Pick up coins placed in pocket of pants<br>7. Practice stacking checkers or playing checkers<br>8. Practice picking up beans<br>9. Place pegs on board using tweezers<br>10. Pick up cotton balls<br>11. Pick up small objects such as pebbles, buttons, paper clips using tweezers.<br>12. Pinch clothespins onto rim of coffee can using thumb	1. Practice tracing<br>2. Practice drawing shapes<br>3. Practice writing<br>4. Practice drawing with a guide (ruler or stencil)<br>5. Practice using fork or spoon during feeding

Refined Hand Movement	In-Hand Manipulation Activities
1. Practice buttoning/unbuttoning with dressing boards<br>2. Sort, clip and fold paper<br>3. Practice opening & closing locks on OT practice board.<br>4. Screw/unscrew nuts and bolts in varying sizes<br>5. Use Valpar 9 to screw/unscrew / 1/4 inch diameter dowels<br>6. Practice typing the home row<br>7. Use affected hand to practice typing words that are only used by one hand. For example: RIGHT: you, mill, pill, nil, him, no<br>LEFT: dear, as, tear, bed, tease, cave, rave, save, rat, pop, kill.<br>8. Practice two handed typing exercise<br>9. On a key ring with multiple keys. Use only one hand to try each key on a door lock.<br>10. Text message a friend or family member using alternating fingers on your weak hand for each word. Stabilize the phone (on a table if needed). Also with strong hand.<br>11. Walk a balloon up and down the wall with your fingertips. Over time and with practice, gradually decrease to smaller sized balls, working down to the size of a ping pong ball.<br>12. Tear a sheet of scrap paper into small pieces and roll the pieces into small wads. Practice flicking the paper wads across the table, alternating fingers<br>13. Wrap a present<br>14. Wind a ball of yarn. When this becomes easy, wind thread around s spool pinching first with index then middle, ring, and pinky fingers.<br>15. Flip marbles or safety pins with fingers.<br>16. Cut out pictures from magazines.<br>17. Using assorted Tupperware types of containers, put on and take off lids.<br>18. Practice using the calculator with one or more of the affected fingers pushing the keys. Start with a large calculator and progress to a small one.<br>19. Practice a computer game using the mouse with the affected hand.<br>20. Practice tying a knot (can start at tabletop and then progress to tying shoe)	1. Practice picking up coin(s) from pocket of pants<br>2. Move coin(s) from fingertips to palm<br>3. Pick up coin(s) from table and hold in palm of hand. Feed the coins back out to thumb and index finger and place on table.<br>4. Practice moving a pen or other object at fingertips<br>5. Practice twirling a pencil<br>6. Rotate a paperclip at fingertips<br>7. Practice changing position of spoon, knife or fork in hand<br>8. Practice finding specific keys from a key ring with multiple keys<br>9. Train release of single object from hand which is holding more than one object<br>10. Take a handful of coins, buttons, ect. Drop them one by one on the table. Then pick them up one by one. See how many you can pick up in one hand before dropping any.<br>11. Pick up pencil and roll it between thumb and fingers<br>12. Assemble and disassemble various sizes of nuts and bolts<br>13. Any of the above activities without vision<br>14. Any of the above with increasing speed

Meaningful Activities of Daily Living

The Canadian Occupational Performance Measure (COPM) was used to identify meaningful activities of daily living to incorporate into therapy sessions and the client was asked to select up to 5 goals. These goals and activities included use of the affected upper extremity. Examples include handwriting, using a smart phone or tablet, using a computer, manipulating money and items in a wallet/purse, cutting food, manipulating tools, playing keyboard, manipulating/fastening jewelry, cooking and grooming.

TOTAL REPS: ________

TOTAL MINS: ________
